# GERIATRIC AND EXTENDED CARE TRAJECTORIES AND TRANSITIONS AMONG VETERANS WITH LONG-TERM CARE NEEDS

**DOI:** 10.1093/geroni/igac059.174

**Published:** 2022-12-20

**Authors:** Erin Bouldin, Benjamin Brintz, Jared Hansen, Randall Rupper, Rachel Brenner, Orna Intrator, Bruce Kinosian, Mary Jo Pugh

**Affiliations:** University of Utah, Salt Lake City, Utah, United States; VA Salt Lake City Health Care System, Salt Lake City, Utah, United States; VA Salt Lake City, Salt Lake City, Utah, United States; University of Utah, Salt Lake City, Utah, United States; University of Utah, Salt Lake City, Utah, United States; Canandaigua VA Medical Center, Canandaigua, New York, United States; University of pennsylvania, Philadelphia, Pennsylvania, United States; University of Utah, Salt Lake City, Utah, United States

## Abstract

We aimed to identify clusters of geriatric and extended care services used by Veterans, describe transitions between clusters, and identify factors influencing transition. We explored services across the continuum of care from clinic to home-based and institutional care services. Analyses include 104,837 Veterans Health Administration (VHA) patients 65 years and older, and used VHA and Centers for Medicare & Medicaid Services (CMS) data from FY15-FY17. Using latent class and latent transition analyses, we identified 5 latent classes: 1) No Services, 2) CMS Services, 3) Home-Centered Interdisciplinary Care, 4) Personal Care Services, and 5) VHA-CMS Care Continuum. Veterans most commonly transitioned from the CMS Care Continuum class to No Services. Veterans also frequently transitioned into the VHA-CMS Care Continuum class over time. Identifying service patterns can inform service delivery, program development, and future resource allocation to better meet aging Veterans’ needs.

